# JIB-04 Has Broad-Spectrum Antiviral Activity and Inhibits SARS-CoV-2 Replication and Coronavirus Pathogenesis

**DOI:** 10.1128/mbio.03377-21

**Published:** 2022-01-18

**Authors:** Juhee Son, Shimeng Huang, Qiru Zeng, Traci L. Bricker, James Brett Case, Jinzhu Zhou, Ruochen Zang, Zhuoming Liu, Xinjian Chang, Tamarand L. Darling, Jian Xu, Houda H. Harastani, Lu Chen, Maria Florencia Gomez Castro, Yongxiang Zhao, Hinissan P. Kohio, Gaopeng Hou, Baochao Fan, Beibei Niu, Rongli Guo, Paul W. Rothlauf, Adam L. Bailey, Xin Wang, Pei-Yong Shi, Elisabeth D. Martinez, Steven L. Brody, Sean P. J. Whelan, Michael S. Diamond, Adrianus C. M. Boon, Bin Li, Siyuan Ding

**Affiliations:** a Department of Molecular Microbiology, Washington University School of Medicine, St. Louis, Missouri, USA; b Program in Molecular Cell Biology, Washington University in St. Louis grid.471404.2grid.4367.6 , St. Louis, Missouri, USA; c Institute of Veterinary Medicine, Jiangsu Academy of Agricultural Sciences, Jiangsu Key Laboratory for Food Quality and Safety-State Key Laboratory Cultivation, Base of Ministry of Science and Technology, Nanjing, China; d Department of Medicine, Division of Infectious Diseases, Washington University School of Medicine, St. Louis, Missouri, USA; e Key Laboratory of Marine Drugs, Ministry of Education, Ocean University of China, Qingdao, China; f Division of Pulmonary and Critical Care Medicine, Department of Medicine, Washington University School of Medicine, St. Louis, Missouri, USA; g National Center for Advancing Translational Sciences, National Institutes of Health, Rockville, Maryland, USA; h Program in Virology, Harvard Medical School, Boston, Massachusetts, USA; i Department of Pathology and Immunology, Washington University School of Medicine, St. Louis, Missouri, USA; j Department of Biochemistry and Molecular Biology, University of Texas Medical Branch, Galveston, Texas, USA; k Department of Pharmacology, UT Southwestern Medical Center, Dallas, Texas, USA; Boston University School of Medicine

**Keywords:** JIB-04, SARS-CoV-2, antiviral agents, coronavirus

## Abstract

Pathogenic coronaviruses are a major threat to global public health. Here, using a recombinant reporter virus-based compound screening approach, we identified small-molecule inhibitors that potently block the replication of severe acute respiratory syndrome virus 2 (SARS-CoV-2). Among them, JIB-04 inhibited SARS-CoV-2 replication in Vero E6 cells with a 50% effective concentration of 695 nM, with a specificity index of greater than 1,000. JIB-04 showed *in vitro* antiviral activity in multiple cell types, including primary human bronchial epithelial cells, against several DNA and RNA viruses, including porcine coronavirus transmissible gastroenteritis virus. In an *in vivo* porcine model of coronavirus infection, administration of JIB-04 reduced virus infection and associated tissue pathology, which resulted in improved weight gain and survival. These results highlight the potential utility of JIB-04 as an antiviral agent against SARS-CoV-2 and other viral pathogens.

## INTRODUCTION

The coronavirus disease 2019 (COVID-19) pandemic has caused unprecedented global morbidity, mortality, and socioeconomic destabilization. Thus, there is an urgent need to develop safe and effective countermeasures to combat the disease beyond vaccine protection and provide immediate treatment. Multiple efforts are under way to identify candidate drugs that inhibit the replication of severe acute respiratory syndrome virus 2 (SARS-CoV-2) (1–5), the cause of COVID-19 (6, 7). So far, several small-molecule inhibitors that interfere with SARS-CoV-2 cell entry have been identified, including transmembrane serine protease inhibitors camostat (8) and nafamostat (9) and endosomal inhibitors including chloroquine and its derivatives (9), E-64d (8), apilimod (10), and 25-hydroxycholesterol (11). Drug screens and structural studies also revealed compounds that target the viral enzymes of SARS-CoV-2, namely, the RNA-dependent RNA polymerase (12–16) and the main protease (M^pro^; also known as 3CL^pro^) (15, 17–19). Most recently, molnupiravir, which is a prodrug for the nucleoside analog and interferes with SARS-CoV-2 replication, has been approved for use in the United Kingdom (20–22). Here, utilizing fluorescent SARS-CoV-2 and an imaging-based screen approach, we identified several known and previously unknown antiviral compounds that inhibit SARS-CoV-2 replication.

## RESULTS

**FIG 1:**
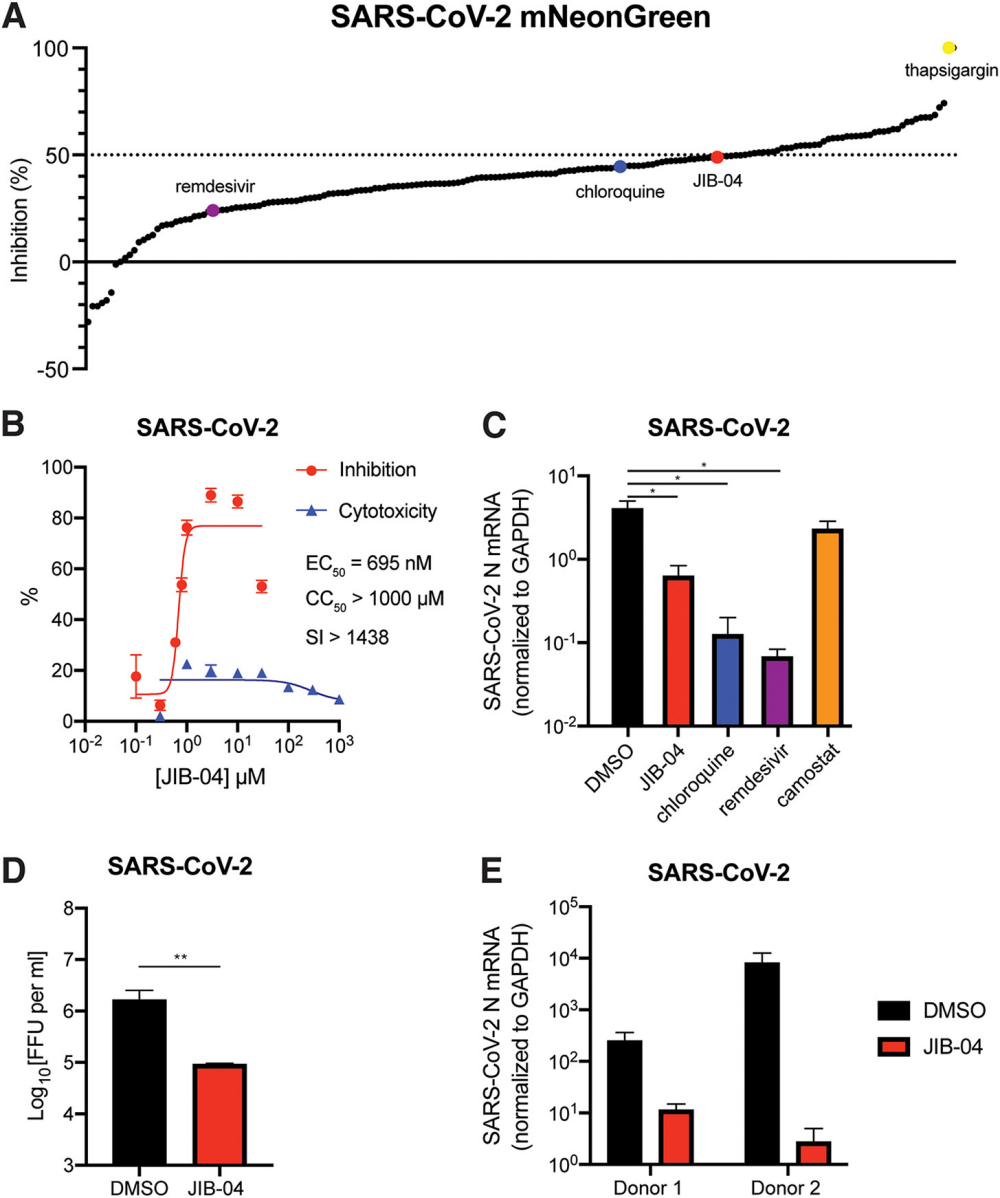
JIB-04 inhibits SARS-CoV-2 replication. (A) Small-molecule inhibitor screen. Vero E6 cells were treated with individual compounds (listed in Table S1) at 10 μM for 1 h and infected with SARS-CoV-2-mNeonGreen (MOI, 0.5). At 24 hpi, cells were fixed and nuclei were stained by Hoechst 33342. The intensities of mNeonGreen and Hoechst were quantified using a Typhoon biomolecular imager and a Cytation plate reader, respectively. The ratio of mNeonGreen and Hoechst is plotted as a percentage of inhibition. (B) Dose-response curve of wild-type SARS-CoV-2 replication with JIB-04 treatment. Vero E6 cells were treated with JIB-04 for 1 h and infected with a clinical isolate of SARS-CoV-2 (MOI, 0.5). S protein levels were quantified at 24 hpi based on immunofluorescence. For CC_50_ measurement, cells were treated with JIB-04 at 0.3 μM to 1 mM for 25 h. SI, selectivity index. (C) Intracellular viral RNA levels of Vero E6 cells treated with compounds and infected with wild-type SARS-CoV-2. Cells were treated with JIB-04 (10 μM), chloroquine (10 μM), remdesivir (3 μM), or camostat mesylate (10 μM) for 1 h and infected with a clinical isolate of SARS-CoV-2 (MOI, 0.5). SARS-CoV-2 N RNA levels at 24 hpi were measured by RT-qPCR. (D) Infectious titers of supernatants in cell culture treated with compounds and infected with wild-type SARS-CoV-2. Vero E6 cells were treated with DMSO (mock) or JIB-04 (10 μM for 1 h) and infected with a clinical isolate of SARS-CoV-2 (MOI, 0.5). Supernatants were harvested at 24 hpi, made into 10-fold serial dilutions, and inoculated into Vero E6 cells. After overnight incubation, infectious titers of supernatants were determined by visualizing the foci using anti-SARS-CoV-2 nucleocapsid protein antibody. FFU, focus-forming units. (E) Intracellular viral RNA levels of primary human airway epithelial cells treated with compounds and infected with wild-type SARS-CoV-2. Primary human bronchial epithelial cells from two different donors, cultured at the air-liquid interface on Transwell membranes, were treated with DMSO (control) or JIB-04 (10 μM) and infected with a clinical isolate of SARS-CoV-2 (MOI, 1). SARS-CoV-2 N RNA levels at 24 hpi were measured by RT-qPCR. For all panels except panel A, experiments were repeated at least three times with similar results. Panel A was performed once with raw data included in Data Set S1. Data are represented as means ± SEM. Statistical significance is from pooled data of the multiple independent experiments (*, *P* ≤ 0.05; **, *P* ≤ 0.01).

To identify small molecules with anti-SARS-CoV-2 activity, we performed a screen using a recombinant SARS-CoV-2 that encoded mNeonGreen as a reporter of infection (23) and an in-house collection of ∼200 compounds that comprised FDA-approved drugs, well-defined broad-spectrum antiviral agents, and investigational new drugs. We identified 157 compounds that had greater antiviral efficacy (>44.8% inhibition) than either chloroquine or remdesivir against SARS-CoV-2 replication in Vero E6 cells (Fig. 1A; see also Data Set S1 in the supplemental material). One of these drugs was a pan-Jumonji histone demethylase inhibitor, 5-chloro-N-[(E)-[phenyl(pyridin-2-yl)methylidene]amino]pyridin-2-amine (JIB-04 E-isomer) (24) (Fig. S1A). We selected JIB-04 E-isomer (JIB-04, unless noted otherwise) for further characterization because several histone demethylases were recently discovered as SARS-CoV-2 host dependency factors (25–27), and JIB-04 has not been reported as an antiviral molecule, although it has established antitumor activity (24, 28–31).

10.1128/mbio.03377-21.1 FIG S1 JIB-04 inhibits SARS-CoV-2 replication. (A) Chemical structures of JIB-04 E-isomer from ChemSpider database. (B) Representative images of Vero E6 cells infected by SARS-CoV-2-mNeonGreen (MOI, 0.5) at 24 hpi in Fig. 1A. Download FIG S1, TIF file, 1.3 MB. Copyright © 2022 Son et al. 2022 Son et al. https://creativecommons.org/licenses/by/4.0/ This content is distributed under the terms of the Creative Commons Attribution 4.0 International license.

10.1128/mbio.03377-21.9 DATA SET S1 Inhibitor screen raw data. Download Data Set S1, XLSX file, 0.03 MB. Copyright © 2022 Son et al. 2022 Son et al. https://creativecommons.org/licenses/by/4.0/ This content is distributed under the terms of the Creative Commons Attribution 4.0 International license.

10.1128/mbio.03377-21.6 TABLE S1 List of chemicals used in the anti-SARS-CoV-2 compound screen. Download Table S1, XLSX file, 0.01 MB. Copyright © 2022 Son et al. 2022 Son et al. https://creativecommons.org/licenses/by/4.0/ This content is distributed under the terms of the Creative Commons Attribution 4.0 International license.

We tested whether JIB-04 treatment could inhibit replication of a clinical isolate of SARS-CoV-2 (2019-nCoV/USA-WA1/2020 strain). Viral antigen staining showed that a 1-h pretreatment with JIB-04 suppressed SARS-CoV-2 infection in Vero E6 cells with a 50% effective concentration (EC_50_) value of 695 nM (95% confidence interval of 567 to 822 nM) (Fig. 1B, Fig. S1B). Cell viability did not fall below 50% even at 1 mM JIB-04 treatment, making the selectivity index of JIB-04 higher than 1,000. JIB-04 also significantly reduced the intracellular SARS-CoV-2 RNA levels, which was not seen when cells were treated with a TMPRSS2 serine protease inhibitor, camostat mesylate (32) (Fig. 1C), implying a distinct mechanism of action. The infectious virus titers of SARS-CoV-2 also were significantly reduced by JIB-04 treatment (Fig. 1D). We further tested the antiviral efficacy of JIB-04 against SARS-CoV-2 in primary human bronchial epithelial cells, well-characterized *in vivo* targets of SARS-CoV-2 (33) (Fig. 1E). SARS-CoV-2 infection of differentiated primary human bronchial epithelium in air-liquid interface culture was substantially inhibited by JIB-04, demonstrating an antiviral effect in more physiologically relevant human airway epithelial cells.

**FIG 2:**
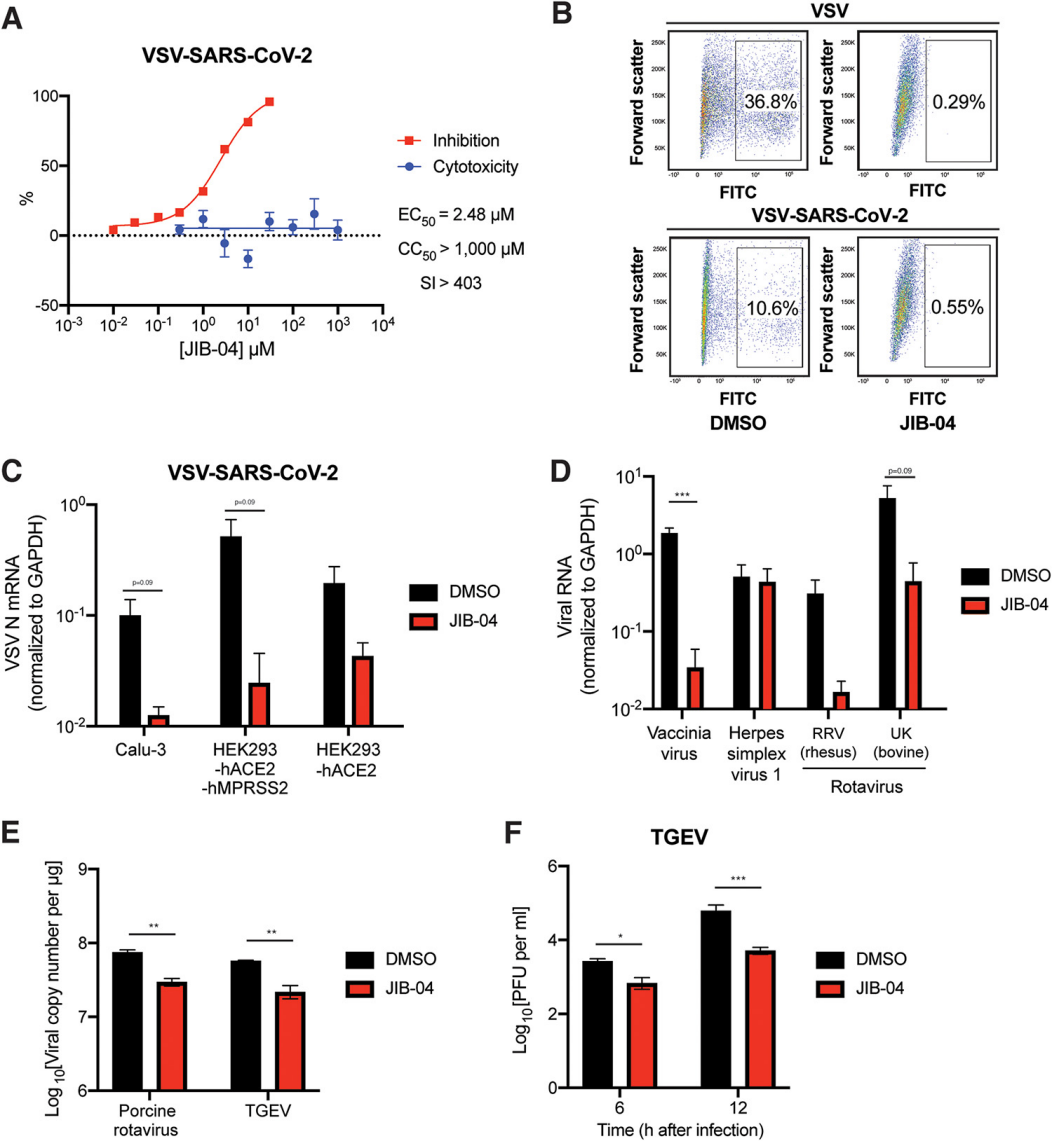
JIB-04 broadly inhibits DNA and RNA viruses in different cell types. (A) Dose-response analysis of VSV-SARS-CoV-2 replication and cytotoxicity with JIB-04 treatment. For EC_50_ measurement, MA104 cells were treated with compounds at 0.01 to 30 μM for 1 h and infected with VSV-SARS-CoV-2 (MOI, 3) for 24 h. For CC_50_ measurement, cells were treated with compounds at 0.1 μM to 3 mM for 25 h. SI, selectivity index. (B) Virus infectivity following JIB-04 treatment. Vero E6-hTMPRSS2 cells were treated with compounds (10 μM) for 1 h and infected with VSV or VSV-SARS-CoV-2 (MOI, 3). At 6 hpi, percentages of GFP-positive cells were quantified by flow cytometry. FITC, fluorescein isothiocyanate. (C) Intracellular viral RNA levels following JIB-04 treatment in different cell types. Calu-3, HEK293-hACE2, and HEK293-hACE2-hTMPRSS2 cells were treated with compounds (10 μM) for 1 h and infected with VSV-SARS-CoV-2 (MOI, 1). VSV RNA levels at 24 hpi were measured by RT-qPCR. (D) Intracellular viral RNA levels following JIB-04 treatment. MA104 cells were treated with compounds (10 μM) for 1 h and infected with vaccinia virus, herpes simplex virus-1, or rotavirus (RRV and UK strains) (MOI, 1). Viral RNA levels at 24 hpi were measured by RT-qPCR for VACV B10R, HSV-1 ICP-27, and RV NSP5, respectively. (E) Viral RNA copy numbers following JIB-04 treatment. HEK293 cells were treated with JIB-04 (10 μM) for 6 h and infected with porcine rotavirus (MOI, 0.01) for 6 h. ST cells were treated with JIB-04 (10 μM) for 12 h and infected with transmissible gastroenteritis virus (TGEV) (MOI, 0.01) for 12 h. Viral RNA copy numbers were measured by RT-qPCR. (F) TGEV titers in the cell supernatant with JIB-04 treatment. ST cells were treated with JIB-04 (10 μM) for 12 h and infected with TGEV (MOI, 0.01). Virus titers at 6 and 12 hpi were measured by plaque assays. For all panels except panels A and B, all experiments were repeated at least three times with similar results. The inhibition assay shown in panel A was performed once and cytotoxicity assay was performed in triplicates. The experiment shown in panel B was performed once. Data are represented as means ± SEM. Statistical significance is from pooled data of the multiple independent experiments (*, *P* ≤ 0.05; **, *P* ≤ 0.01; ***, *P* ≤ 0.001).

To examine whether JIB-04 targets SARS-CoV-2 spike (S) protein-mediated entry or other postentry pathways (e.g., translation, replication, or assembly) shared between SARS-CoV-2 and other viruses, we tested JIB-04 against vesicular stomatitis virus (VSV) that expresses enhanced green fluorescent protein (eGFP) as a marker of infection (34) and a replication-competent chimeric VSV that harbors SARS-CoV-2 S protein in lieu of the native glycoprotein (G) and also expresses eGFP (VSV-SARS-CoV-2) (35). JIB-04 suppressed replication of both viruses in MA104 and Vero E6-hTMPRSS2 cells (Fig. 2A and B). Flow cytometry analysis of cells at 6 h postinfection (hpi) revealed a reduction in eGFP expression, demonstrating that the inhibitory effect of JIB-04 occurs during early steps of virus infection (Fig. 2B). Virus-infected cells also showed less GFP intensity with JIB-04 treatment (Fig. S2A). JIB-04 inhibited VSV-SARS-CoV-2 infection in a dose-dependent manner without apparent cytotoxicity (Fig. 2A, Fig. S2B), suggesting that the blockade is not at the entry step. Inhibition was more evident when cells were inoculated with virus at a low multiplicity of infection (MOI) (Fig. S2C). At 30 μM, JIB-04 treatment resulted in a 100-fold reduction of intracellular VSV-SARS-CoV-2 RNA levels (Fig. S2D). We also confirmed the inhibitory effect of JIB-04 on VSV-SARS-CoV-2 infection in human lung epithelial cell line Calu-3 (8, 20) (Fig. 2C). VSV-SARS-CoV-2 replication also was inhibited by JIB-04 in HEK293 cells ectopically expressing human ACE2, an entry receptor for SARS-CoV-2 (8), either with or without ectopic hTMPRSS2 expression.

10.1128/mbio.03377-21.2 FIG S2 JIB-04 inhibits the replication of multiple viruses. (A) Mean fluorescence intensity of GFP positive cells in Fig. 2B was quantified by flow cytometry. (B) Dose-response analysis of VSV-SARS-CoV-2 replication with JIB-04 treatment. MA104 cells were treated with JIB-04 at the indicated concentrations for 1 h and infected with VSV-SARS-CoV-2 (MOI, 3). At 24 hpi, images of GFP-positive infected cells were acquired using an ECHO fluorescence microscope. (C) Same as panel B, except that cells were infected with an MOI of 0.1. (D) Dose-response analysis of intracellular viral RNA levels with JIB-04 or chloroquine treatment. MA104 cells were treated with compounds at 0.1 to 30 μM for 1 h and infected with VSV-SARS-CoV-2 (MOI, 3). VSV RNA levels at 24 hpi were measured by RT-qPCR. (E) Western blot analysis of RV antigen VP6 levels with JIB-04 treatment. HEK293 cells were treated with JIB-04 at 1, 5, or 10 μM for 6 h and infected with porcine RV (MOI, 0.01) for 12 h. GAPDH was used as a loading control. All experiments were repeated at least three times with similar results. Data are represented as means ± SEM. Download FIG S2, TIF file, 2.7 MB. Copyright © 2022 Son et al. 2022 Son et al. https://creativecommons.org/licenses/by/4.0/ This content is distributed under the terms of the Creative Commons Attribution 4.0 International license.

We next evaluated the antiviral activity of JIB-04 against other viruses. Although JIB-04 did not diminish replication of herpes simplex virus 1, it inhibited the replication of vaccinia virus, another DNA virus, and several strains of rotavirus (RV), a double-stranded RNA virus (Fig. 2D and E). JIB-04 also inhibited the replication of transmissible gastroenteritis virus (TGEV) (Fig. 2E and F, Fig. S2E), a porcine coronavirus that infects the small intestine of pigs and causes lethal diarrhea (36). These observations indicate that the antiviral effect of JIB-04 is not limited to single-stranded RNA viruses in cell culture.

**FIG 3:**
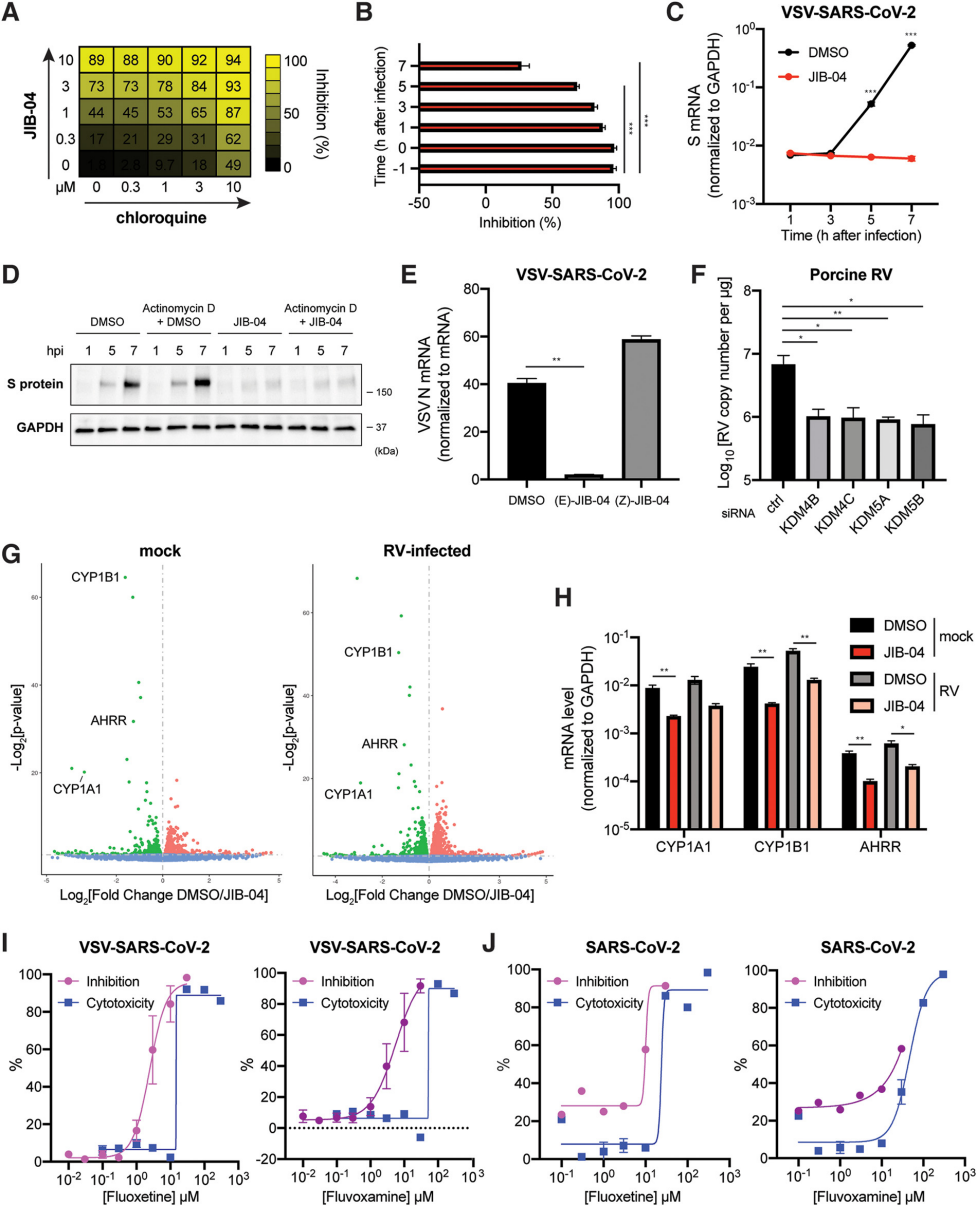
JIB-04 exhibits distinct postentry antiviral mechanisms. (A) Drug combination dose-response matrix and VSV-SARS-CoV-2 replication. MA104 cells were treated with JIB-04 and chloroquine for 1 h and infected with VSV-SARS-CoV-2 (MOI, 3). GFP signals at 24 hpi were quantified to calculate the percentage of inhibition. (B) Time of compound addition and VSV-SARS-CoV-2 replication. MA104 cells were treated with JIB-04 (10 μM) at indicated time points relative to VSV-SARS-CoV-2 infection (MOI, 3; 0 hpi). GFP signals at 8 hpi were quantified to calculate the percentage of inhibition. (C) Intracellular SARS-CoV-2S RNA levels with JIB-04 treatment. MA104 cells were treated with JIB-04 (10 μM) for 1 h and infected with VSV-SARS-CoV-2 (MOI, 1) for 1, 3, 5, and 7 h. S RNA levels were measured by RT-qPCR. (D) Western blot analysis of SARS-CoV-2 S protein levels with JIB-04 treatment. MA104 cells were treated with JIB-04 (10 μM) for 1 h and infected with VSV-SARS-CoV-2 (MOI, 1) for 1, 5, and 7 h. For actinomycin D, 10 μg/ml actinomycin D was added to the medium 15 min before DMSO or JIB-04 treatment. (E) Intracellular VSV-SARS-CoV-2 RNA levels of cells treated with JIB-04 isomers. MA104 cells were treated with JIB-04 E or Z isomer (10 μM) for 1 h and infected with VSV-SARS-CoV-2 (MOI, 1). VSV N levels at 24 hpi were measured by RT-qPCR. (F) Histone demethylase siRNA knockdown and RV replication. HEK293 cells were transfected with scrambled siRNA or siRNA targeting indicated histone demethylases for 48 h and infected with porcine RV (MOI, 0.01). Viral RNA copy numbers at 12 hpi were quantified by RT-qPCR. (G) Volcano plot of differentially expressed transcripts with JIB-04 treatment and RV infection. HEK293 cells were treated with DMSO or JIB-04 (10 μM) for 12 h and mock infected (left) or infected with porcine RV (MOI, 0.01) (right) for another 12 h. Red dots represent upregulated genes and green dots represent downregulated genes in JIB-04 treated cells. (H) Expression of three top genes in panel G with JIB-04 treatment. HEK293 cells were treated with JIB-04 (10 μM) for 12 h and mock infected or infected with porcine RV (MOI, 0.01) for 12 h. mRNA levels of *CYP1A1*, *CYP1B1*, and *AHRR* at 12 hpi were measured by RT-qPCR. (I) Dose-response analysis of VSV-SARS-CoV-2 replication with fluoxetine or fluvoxamine treatment. MA104 cells were treated with compounds at 0.01 to 30 μM for 1 h and infected with VSV-SARS-CoV-2 (MOI, 3). GFP signals at 24 hpi were quantified to calculate the percentage of inhibition. For CC_50_ measurement, cells were treated with compounds at 0.1 μM to 300 μM for 25 h. (J) Dose-response analysis of wild-type SARS-CoV-2 replication with fluoxetine or fluvoxamine treatment. Vero E6 cells were treated with compounds for 1 h and infected with a clinical isolate of SARS-CoV-2 (MOI, 0.5). S protein levels at 24 hpi were quantified based on immunofluorescence. For CC_50_ measurement, cells were treated with compounds at 0.1 μM to 300 μM for 25 h. For all panels except panels A and J, experiments were repeated at least three times with similar results. The experiment shown in panel A was performed twice. The inhibition assay shown in panel J was performed once, and the cytotoxicity assay was performed in triplicates. Data are represented as means ± SEM. Statistical significance is from pooled data of the multiple independent experiments (*, *P* ≤ 0.05; **, *P* ≤ 0.01; ***, *P* ≤ 0.001).

We next sought to understand the mechanisms of antiviral activity of JIB-04. Although JIB-04 was previously connected to interferon (IFN) and autophagy activation (24, 37), the antiviral activity that we observed was independent of these pathways. JIB-04 treatment did not lead to the induction of IFN and IFN-stimulated gene expression or the formation of LC3-positive punctate structures (Fig. S3A and B). To explore the mechanisms of antiviral action, we tested the synergy of drug combinations. When JIB-04 was assessed for the combinatorial activity with chloroquine in MA104 cells by SynergyFinder 2.0 (38) (Fig. 3A), it showed a synergy δ-score of 10.782, 7.93, 13.854, and 8.106 for four different existing reference models: highest single agent, Bliss independence, Loewe additivity, and zero interaction potency, respectively. These results fulfill the synergy criteria for all four models, showing that JIB-04 likely exerts a synergistic antiviral effect with chloroquine (39). No obvious cytotoxicity was observed for the combined treatment of JIB-04 and chloroquine, even at the highest concentrations tested (10 μM) (Fig. S3C). A combination of JIB-04 and camostat also was synergistically antiviral in Calu-3 cells (Fig. S3D), indicating that JIB-04 targets a different pathway than chloroquine or camostat, which target viral entry.

10.1128/mbio.03377-21.3 FIG S3 Inhibition or knockdown of specific KDM histone demethylases inhibits virus replication. (A) Expression of IFN and IFN-stimulated genes with JIB-04 treatment. HEK293 cells were treated with JIB-04 (3 μM) or transfected with low-molecular-weight poly(I:C) (100 ng/ml) for 24 h. mRNA levels of IFNL3 and CXCL10 were measured by RT-qPCR. (B) Autophagy formation with compound treatment. HEK293 cells were transfected with EGFP-LC3 plasmid for 24 h and treated with rapamycin (100 nM) or JIB-04 (3 μM) for another 18 h. GFP-positive punctate structures indicate autophagy activation. Scale bar, 20 μm. (C) Drug combination cytotoxicity matrix. MA104 cells were treated with JIB-04 and chloroquine at indicated concentrations for 25 h. Cell viability was measured by CCK-8 assay. (D) Intracellular viral RNA levels with JIB-04 and camostat treatment. Calu-3 cells were treated with compounds (10 μM) for 1 h and infected with VSV-SARS-CoV-2 (MOI, 3). VSV RNA levels at 24 hpi were measured by RT-qPCR. (E) siRNA-mediated knockdown of JIB-04 target histone demethylases. HEK293 cells were transfected with scrambled siRNA or siRNA targeting indicated histone demethylases for 48 h. mRNA levels of indicated histone demethylases were measured by RT-qPCR. (F) Western blot analysis of RV antigen VP6 levels in cells with histone demethylase siRNA knockdown. HEK293 cells were transfected with scrambled siRNA or siRNA targeting indicated histone demethylases for 48 h and infected with porcine RV (MOI, 0.01) for 12 h. (G) Pathway enrichment analysis of gene expression regulated by JIB-04 treatment. Downregulated genes in Fig. 3F with *P* values of <1e−10 were analyzed by DAVID functional annotation. For all panels except panel B, experiments were repeated at least three times with similar results. Experiments for panel B was performed twice. Data are represented as means ± SEM. Statistical significance is from pooled data of the multiple independent experiments (*, *P* ≤ 0.05; **, *P* ≤ 0.01; ***, *P* ≤ 0.001). Download FIG S3, TIF file, 2.7 MB. Copyright © 2022 Son et al. 2022 Son et al. https://creativecommons.org/licenses/by/4.0/ This content is distributed under the terms of the Creative Commons Attribution 4.0 International license.

A possible antiviral role for JIB-04 at a postentry step was supported by the time-of-addition experiments (Fig. 3B). Simultaneous treatment of JIB-04 with the virus infection achieved a similar level of inhibitory activity as that at 1 h pretreatment (Fig. 3B). The addition of compound at 3 hpi still resulted in an inhibitory activity of about 80% (Fig. 3B). We next examined the viral mRNA transcription, an early event after viral entry, as well as its translation to evaluate the step(s) affected by JIB-04. A 1 h pretreatment of cells with JIB-04 reduced SARS-CoV-2 S mRNA transcription following VSV-SARS-CoV-2 infection (Fig. 3C) and translation of newly synthesized S protein, which could not be achieved with actinomycin D treatment (Fig. 3D). These results suggest that JIB-04 represses virus replication by interfering with the viral RNA transcription or stability.

We also assessed whether the antiviral activity of JIB-04 is linked to its epigenetic modulatory action. Unlike its E-isomer, the Z-isomer of JIB-04 does not inhibit histone demethylases at similar doses (24). When we compared the antiviral efficacy of these two JIB-04 isomers against VSV-SARS-CoV-2 in MA104 cells, the Z-isomer did not inhibit the replication of virus (Fig. 3E). The disparity between the isomers implies that the antiviral mechanisms of JIB-04 likely involves the inhibition of histone demethylases. To examine the cellular pathways modulated by JIB-04, we performed small interfering RNA (siRNA)-mediated knockdown of JIB-04 cellular targets (i.e., histone demethylase KDM4B, KDM4C, KDM5A, or KDM5B [24]). Knockdown of each gene successfully recapitulated the antiviral effect of JIB-04 (Fig. 3F, Fig. S3E and F). These results led us to hypothesize that JIB-04 treatment promoted H3K9 and H3K27 methylation and silenced expression of a subset of genes, triggering the antiviral effect. To identify potential target genes, we performed RNA sequencing on the cells pretreated with vehicle or JIB-04 with or without virus infection (Fig. 3G). Pathway analysis revealed dampened metabolic signaling pathways, such as the cytochrome P450 system in JIB-04 treated cells (Fig. S3G). Specifically, JIB-04 treatment downregulated two cytochrome P450 enzymes, *CYP1A1* and *CYP1B1*, and an aryl hydrocarbon receptor repressor (*AHRR*), which represses the transactivator of *CYP1A1* and *CYP1B1* (40). We validated by reverse transcription-quantitative PCR (RT-qPCR) that JIB-04 treatment reduced *CYP1A1*, *CYP1B1*, and *AHRR* mRNA levels by 4- to 6-fold (Fig. 3H). To explore the pharmacological utility of this finding, we tested the antiviral activity of cytochrome P450 enzyme inhibitors fluoxetine and fluvoxamine (41). Both compounds inhibited the replication of VSV-SARS-CoV-2 (Fig. 3I) as well as wild-type SARS-CoV-2 (Fig. 3J).

**FIG 4:**
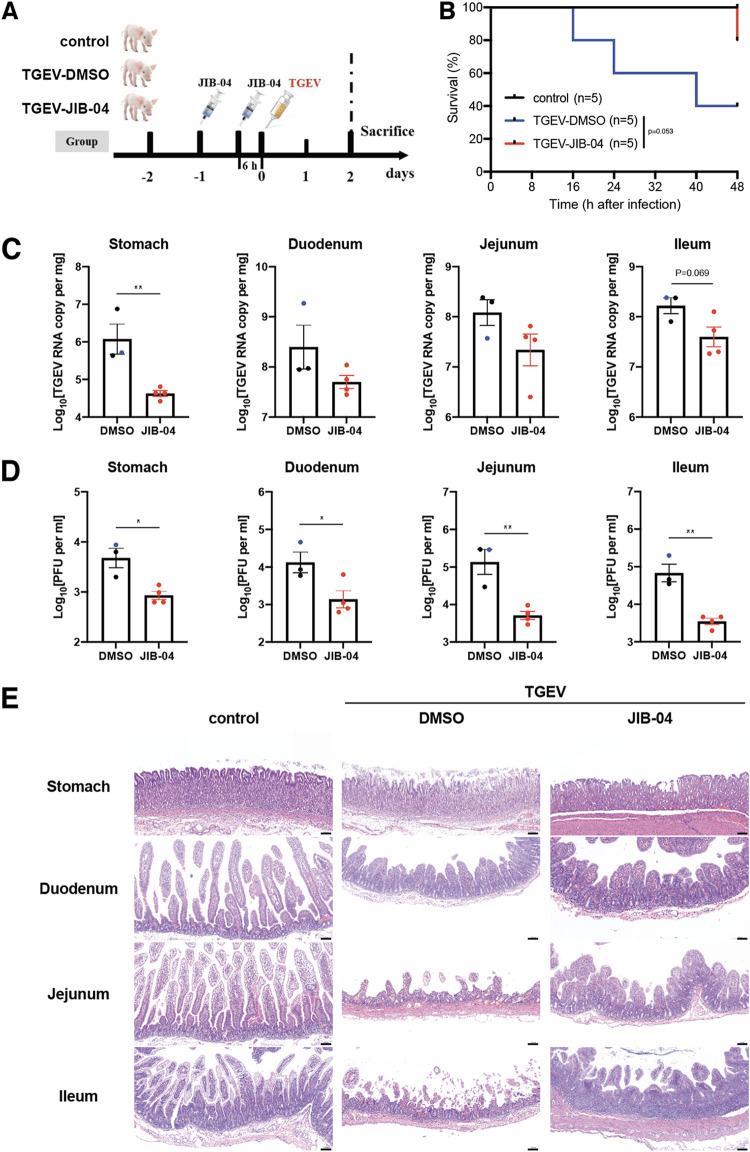
Prophylactic administration of JIB-04 suppresses TGEV replication and pathogenesis in pigs. (A) Experimental schemes for testing the prophylactic efficacy of JIB-04 treatment against TGEV challenge in three groups of neonatal pigs. Neonatal pigs were mock infected or infected with 1.2 × 10^7^ PFU of TGEV and intraperitoneally injected with vehicle control DMSO or JIB-04. Control, DMSO injection, mock infection; TGEV-DMSO, DMSO injection, TGEV infection; TGEV-JIB-04, JIB-04 injection, TGEV infection. (B) Survival curve of TGEV-infected pigs with JIB-04 treatment. Survival was monitored every 8 h with data censored at 48 hpi, when all pigs were euthanized. (C) TGEV RNA copy numbers in the gastrointestinal (GI) tract of infected pigs. TGEV-infected pigs were sacrificed at 48 hpi. For the DMSO group, two animals sacrificed at 48 hpi and one that died at 40 hpi (colored in blue) were examined. For the JIB-04 groups, four animals sacrificed at 48 hpi were examined. TGEV genome copy numbers at 48 hpi were quantified by RT-qPCR. (D) Same as panel C, except that virus titers were measured by plaque assays. (E) Hematoxylin and eosin staining of different GI tract sections from pigs sacrificed at 48 hpi. Representative images of 3 animals. Scale bar, 100 μm. Data are represented as means ± SEM. Statistical significance is from pooled data of the multiple independent experiments (*, *P* ≤ 0.05; **, *P* ≤ 0.01).

Given that JIB-04 prevents coronavirus replication *in vitro*, we used a neonatal pig TGEV infection model (42) to evaluate the efficacy of JIB-04 against coronavirus infection *in vivo*. Two-day-old piglets were injected via the intraperitoneal route with JIB-04 twice before the oral inoculation of TGEV (Fig. 4A). We monitored body weight daily and recorded diarrhea development and mortality every 6 h. The animals in the control group lost more weight and had more severe diarrhea than those receiving JIB-04 (Fig. S4A and B). At 2 days postinfection (dpi), 3 of 5 piglets in the control group succumbed to infection compared to 1 out of 5 animals in the JIB-04 group (Fig. 4B). Consistent with our *in vitro* results (Fig. 2E and F), the TGEV burden throughout the gastrointestinal (GI) tract was substantially lower in the JIB-04-treated group (Fig. 4C and D). JIB-04-treated animals also had fewer viral antigen-positive cells in their intestinal epithelium (Fig. S4C) and showed less enteropathy than the control group (Fig. 4E).

10.1128/mbio.03377-21.4 FIG S4 JIB-04 reduces TGEV-induced weight loss and pathogenesis. (A) Weight of pigs in Fig. 4A. The body weight of individual animals was monitored every 24 h. (B) Diarrhea occurrence in pigs in Fig. 4A. Diarrhea severity was scored for the fecal specimens of DMSO or JIB-04-treated, mock-treated, or TGEV-infected animals every 8 h. (C) Immunofluorescence staining of TGEV antigen in different GI tract sections from pigs sacrificed at 48 hpi. Blue, cell nuclei; red, TGEV nucleocapsid protein. Representative images of 3 animals. Scale bar, 100 μm. Download FIG S4, TIF file, 2.3 MB. Copyright © 2022 Son et al. 2022 Son et al. https://creativecommons.org/licenses/by/4.0/ This content is distributed under the terms of the Creative Commons Attribution 4.0 International license.

**FIG 5:**
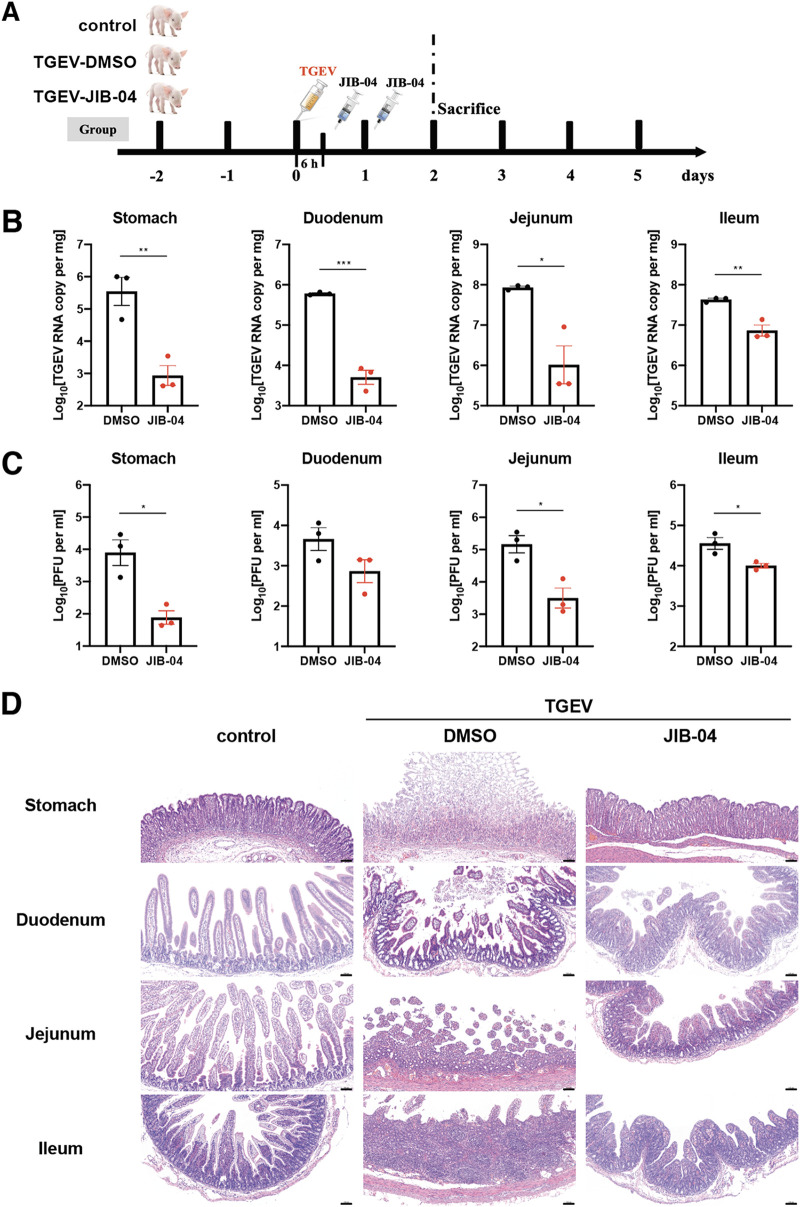
Therapeutic administration of JIB-04 suppresses TGEV replication and pathogenesis in pigs. (A) Experimental schemes for testing the therapeutic efficacy of JIB-04 treatment against TGEV challenge in three groups of neonatal pigs. Neonatal pigs were mock infected or infected with 1.2 × 10^7^ PFU of TGEV and intraperitoneally injected with the vehicle control DMSO or JIB-04. Three animals from each group were sacrificed at 48 hpi for tissue analysis. Control, DMSO injection, mock infection; TGEV-DMSO, DMSO injection, TGEV infection; TGEV-JIB-04, JIB-04 injection, TGEV infection. (B) TGEV RNA copy numbers in the GI tract of infected pigs sacrificed at 48 hpi. TGEV genome copy numbers were quantified by RT-qPCR. (C) Same as panel B except that virus titers were measured by plaque assays. (D) Hematoxylin and eosin staining of different GI tract sections from pigs sacrificed at 48 hpi. Representative images of 3 animals. Scale bar, 100 μm. (E) Data are represented as means ± SEM. Statistical significance is from pooled data of multiple independent experiments (*, *P* ≤ 0.05; **, *P* ≤ 0.01).

We used the same pig TGEV infection model to assess the therapeutic potential of JIB-04 against coronavirus. Two-day-old piglets were injected via the intraperitoneal route with JIB-04 6 h and 24 h after the oral inoculation of TGEV (Fig. 5A). In contrast to prophylactic administration, therapeutic administration of JIB-04 did not improve survival or weight loss and only marginally lessened the diarrhea severity in infected animals (Fig. S5A to C). Nonetheless, the TGEV load was significantly lower in the JIB-treated group throughout the GI tract (Fig. 5B and C). JIB-04 administration also ameliorated the damage of GI epithelium in TGEV-infected animals (Fig. 5D), with fewer viral antigen-positive cells (Fig. S5D). Taken together, our data demonstrate *in vivo* antiviral activity of JIB-04 against a porcine coronavirus.

10.1128/mbio.03377-21.5 FIG S5 JIB-04 reduces TGEV induced pathogenesis. (A) Survival curve of pigs in Fig. 5A. With 3 animals from each group being sacrificed at 48 hpi, survival of remaining animals was monitored every 8 h up to 5 days following infection. (B) Weight of pigs in Fig. 5A. The body weight of individual animals was monitored every 24 h. (C) Diarrhea occurrence in pigs Fig. 5A. Diarrhea severity was scored for the fecal specimens of DMSO- or JIB-04-treated, mock-treated, or TGEV-infected animals every 8 h. (D) Immunofluorescence staining of TGEV antigen in different GI tract sections from pigs sacrificed at 48 hpi. Blue, cell nuclei; red, TGEV nucleocapsid protein. Representative images of 3 animals. Scale bar, 100 μm. Download FIG S5, TIF file, 2.5 MB. Copyright © 2022 Son et al. 2022 Son et al. https://creativecommons.org/licenses/by/4.0/ This content is distributed under the terms of the Creative Commons Attribution 4.0 International license.

## DISCUSSION

Using a repurposed compound screening approach, we identified drugs with reported inhibitory activity against SARS-CoV-2, such as tetrandrine (43) and arbidol (44). We also characterized several small molecules (JIB-04, AG-1478, nigericin, etc.) without known antiviral activity as inhibitors of SARS-CoV-2 infection. While the manuscript was in preparation, another study identified thapsigargin, the compound that showed the highest anti-SARS-CoV-2 activity in our screen, as a broad antiviral against coronavirus (45), which validates our screen approach. Notably, several top-hit compounds in the screen converge on the endosomal trafficking pathway: brefeldin A, concanamycin A, tetrandrine, and U18666A. Furthermore, FTY720 induced formation of enlarged endosome/lysosome structures, resembling one triggered by apilimod treatment (10). This observation is consistent with the property of hTMPRSS2-negative Vero E6 cells we used for screening in which SARS-CoV-2 relies on endosomal machinery for entry (8, 43).

While the window for advancing repurposed drugs against COVID-19 may be closing soon, our results highlight JIB-04 as a potential antiviral agent against SARS-CoV-2 as well as other viral diseases and suggest further evaluation of this drug, which has mainly been associated with its anticancer activities. Another compound in our screen, GSK-J4, also is a histone demethylase inhibitor that targets KDM6B. However, unlike JIB-04, GSK-J4 failed to reduce viral burden in Vero E6 cells upon SARS-CoV-2 infection to an extent comparable to that of chloroquine. Thus, we speculate that there are specific roles played by certain KDM family members in the interactions between the host and SARS-CoV-2.

### Limitations of the study.

We have been cautious about reporting the broad-spectrum antiviral activity of JIB-04. Indeed, we have shown examples of inhibition on viruses from distinct families (double-stranded DNA virus, vaccinia virus; single-stranded positive-sense RNA virus, SARS-CoV-2 and TGEV; single-strand negative-sense RNA virus, VSV and VSV-SARS-CoV-2; double-stranded RNA virus, RV). However, we have yet to test single-stranded DNA viruses or retroviruses. We also did not examine whether JIB-04 has antiviral activity against the newly emerging SARS-CoV-2 variants. We showed that JIB-04 modulates cytochrome P450 genes, and independent targeting of these genes by a well-established selective serotonin uptake inhibitor also inhibited SARS-CoV-2 replication. Nevertheless, we do not know whether modulation of cytochrome P450 genes correlates with transcriptional repression of SARS-CoV-2 RNA that we observed after JIB-04 treatment. It is plausible that JIB-04 induces these two effects separately, which needs to be characterized in future studies. Lastly, although we provided evidence that JIB-04 suppressed coronavirus replication *in vivo* using a porcine TGEV model, enhanced survival or weight loss of animals was observed only in prophylactic but not therapeutic settings, the latter of which would be important for utility in clinical application. TGEV also is an animal coronavirus that targets the enteric rather than the respiratory system. The efficacy of JIB-04 against SARS-CoV-2 should be tested in animal models once pharmacokinetics is established. Finally, while the distinct efficacy of the E versus Z isomers of JIB-04 points to the inhibition of Jumonji demethylases as contributing to the antiviral effects, direct evidence of this mechanism is required to support this conclusion.

## MATERIALS AND METHODS

### Reagents, cells, and viruses. (i) Reagents.

JIB-04 E-isomer was used in *in vitro* assays (99.8% purity; S7281; Selleckchem), JIB-04 E-isomer was used in *in vivo* experiments (HY-13953; Med-ChemExpress), and actinomycin D (A5156; Sigma), fluvoxamine maleate (S1336; Selleckchem), fluoxetine HCl (S1333; Selleckchem), and low-molecular-weight poly(I:C) complexed with LyoVec (tlrl-picwlv; InvivoGen) were used. EGFP-LC3 plasmid was a gift from Christina Stallings at Washington University School of Medicine. pUC19 empty plasmid was used as mock treatment in all transfection experiments. JIB-04 Z-isomer used in control experiments was synthesized as originally described (24).

### (ii) Cells.

Vero E6 cells (CRL-1586; ATCC) and Vero cells (CCL81; ATCC) were cultured in Dulbecco’s modified Eagle’s medium (DMEM) supplemented with 10% fetal bovine serum (FBS), 10 mM HEPES, 1 mM sodium pyruvate, 0.1 mM nonessential amino acids, and 1× penicillin-streptomycin-glutamine. Calu-3 cells (HTB-55; ATCC) and swine ST cells (CRL-1746; ATCC) were cultured in DMEM supplemented with 10% FBS and 1× penicillin-streptomycin-glutamine. HEK293, HEK293-hACE2, and HEK293-hACE2-hTMPRSS2 cells were cultured as previously described (46). MA104 and Vero E6-hTMPRSS2 cells were cultured as before (46). Primary human bronchial epithelial cells (HBECs) were isolated from surgical excess of lungs donated for lung transplantation and anonymized (one female, one male). Isolated basal progenitor cells were seeded on a Transwell (Corning) and differentiated to secretory and multiciliated cell types using air-liquid interface conditions, as previously described (47).

### (iii) Viruses.

Rhesus RV RRV strain, bovine RV UK strain, and porcine RV NJ2012 strain (GenBank accession numbers MT874983–MT874993) were propagated and titrated as before (48). Vaccinia virus MVA strain was used as before (49). HSV-1 syn17^+^ strain was a gift from Ann Arvin at Stanford University. TGEV JS2012 strain was propagated as before (50). TGEV was titrated by serial dilutions in cells in 96-well plates that were seeded at a density of 1 × 10^4^ cells per well. Cytopathic effects were observed at 3 to 7 dpi, and the 50% tissue culture infectious dose (TCID_50_) values were calculated and converted to PFU per ml. A clinical isolate of SARS-CoV-2 (2019-nCoV/USA-WA1/2020 strain) was obtained from the Centers for Disease Control and Prevention. A SARS-CoV-2 mNeonGreen reporter virus was used as previously reported (23). Both the clinical isolate and the mNeonGreen SARS-CoV-2 viruses were propagated in Vero CCL81 cells and titrated by focus-forming assays on Vero E6 cells. Recombinant VSV-eGFP (34) and VSV-eGFP-SARS-CoV-2 were previously described (35). Cells infected with viruses expressing GFP were imaged with an ECHO REVOLVE 4 fluorescence microscope. Plaque assays were performed in MA104 cells seeded in 6-well plates using an adapted version of the rotavirus plaque assay protocol (48).

### Inhibitor screen.

The small-molecule inhibitors used in this study are from an in-house collection and the COVID Box (Medicines for Malaria Venture; www.mmv.org/mmv-open/covid-box). Compound names, vendors, and catalog numbers are listed in Table S1 in the supplemental material. At 24 hpi, cells were fixed in 4% paraformaldehyde (PFA) in phosphate-buffered saline (PBS) and stained with Hoechst 33342. The levels of viral antigens, reflected by mNeonGreen signals, were scanned by Amersham Typhoon 5 (GE). Image background was subtracted using a rolling ball algorithm (radius of 5 pixels). To minimize imaging artifacts and well-to-well variation, we removed the region that fell below the threshold calculated by the Moments algorithm. The number of positive pixels and total intensity (after background subtraction) were computed for each well and log_10_ transformed. The number of cells in each well was quantified based on Hoechst 33342 staining detected by a Cytation 3 imaging reader (BioTek). Image analysis was performed using ImageJ and customized R scripts. The quantification of mNeonGreen and Hoechst 33342 is provided in Data Set S1.

### Cell cytotoxicity assay.

The viability of Vero E6 and MA104 cells after drug treatment was determined using the Cell Counting kit 8 (ab228554; Abcam). Briefly, cells in 96-well plates were treated with drug(s) at desired concentrations at 37°C. After 25 h of incubation, medium was replaced with fresh complete medium with 10 μl of WST-8 solution in each well. The cells were incubated at 37°C for 2 h with protection from light. Absorbance at 460 nm was read using an ELx800 microplate reader (BioTek).

### RNA extraction and RT-qPCR.

Total RNA was extracted from cells using an RNeasy minikit (Qiagen). For spike plasmid transfection experiments, total RNA was extracted using an Aurum total RNA minikit (Bio-Rad) with DNase digestion. cDNA was synthesized with a high-capacity cDNA reverse transcription kit (Thermo Fisher) as previously described (51). qPCR was performed using AriaMX (Agilent) with 12.5 μl of either Power SYBR green master mix or TaqMan master mix (Applied Biosystems) in a 25 μl reaction mixture. Gene expression was normalized to the housekeeping gene glyceraldehyde-3-phosphate dehydrogenase (GAPDH). All SYBR green primers and TaqMan probes used in this study are listed in Table S2.

10.1128/mbio.03377-21.7 TABLE S2 List of qPCR primers and siRNA. Download Table S2, XLSX file, 0.01 MB. Copyright © 2022 Son et al. 2022 Son et al. https://creativecommons.org/licenses/by/4.0/ This content is distributed under the terms of the Creative Commons Attribution 4.0 International license.

### Western blotting.

For Western blotting, cells were lysed in radioimmunoprecipitation assay buffer supplemented with protease inhibitor cocktail and phosphatase inhibitor. Lysates were boiled for 5 min in 1× Laemmli sample buffer (Bio-Rad) containing 5% β-mercaptoethanol. Proteins were resolved in SDS-PAGE and detected as described previously (52) using the following antibodies: GAPDH (631402; BioLegend), RV VP6 (rabbit polyclonal; ABclonal Technology), and SARS-CoV-2 S2 (40592-T62; Sino Biological). Secondary antibodies were either anti-rabbit (7074; Cell Signaling) or anti-mouse (7076; Cell Signaling) immunoglobulin G horseradish peroxidase-linked antibodies. Blots were developed using Clarity ECL substrate (Bio-Rad), and the protein bands were visualized using a Gel Doc XR system (Bio-Rad).

### siRNA transfection.

HEK293 cells were transfected using Lipofectamine RNAiMAX transfection reagent (Thermo Fisher). Cells were harvested at 48 h posttransfection, and knockdown efficiency was determined by RT-qPCR. All siRNAs used in this study were designed and synthesized by GenePharma (Shanghai, China), and the sequences of their siRNAs are listed in Table S2.

### Flow cytometry.

Vero E6-hTMPRSS2 cells were infected with VSV-eGFP or VSV-SARS-CoV-2 at an MOI of 3 for 1 h at 37°C. At 6 hpi, cells were harvested and fixed in 4% PFA in PBS. The percentage of GFP-positive cells and GFP intensity were determined using an LSRFortessa X-20 cell analyzer (BD) and analyzed by FlowJo v10.6.2 (BD).

### RNA-seq.

HEK293 cells were pretreated with JIB-04 (10 μM) for 12 h and mock or porcine RV infected (MOI, 0.01) for another 12 h. Total RNA from cells in triplicate was extracted using an RNeasy minikit (Qiagen). RNA sample quality was measured by both NanoDrop spectrophotometer (Thermo Fisher) and Bioanalyzer 2100 (Agilent). Libraries were sequenced on the Illumina NovaSeq 6000 platform. Differential gene expression analysis was performed using DESeq2. The RNA sequencing (RNA-seq) raw and processed data sets were deposited into the NCBI Gene Expression Omnibus database (GSE156219).

### Pig experiments.

Neonatal male pigs (Landrace × Yorkshire) spontaneously delivered from sows were obtained at birth from a TGEV-free farm in Nanjing. All pigs were confirmed negative for TGEV by RT-PCR and enzyme-linked immunosorbent assay (IDEXX, USA). They were randomly separated into three groups, housed in separate rooms, and fed the same artificial milk substitutes that meet the nutrient and energy recommendations of the National Research Council (NRC; 2012) at the animal facility of the Institute of Veterinary Medicine, Jiangsu Academy of Agricultural Sciences, Nanjing, Jiangsu Province, China.

### (i) Prophylactic administration experiment.

A total 15 of animals were used and divided into three groups: a DMSO administration control group (control, *n* = 5), a DMSO administration and TGEV infection group (TGEV-DMSO, *n* = 5), and a JIB-04 administration and TGEV infection group (TGEV-JIB-04, *n* = 5). Neonatal pigs were intraperitoneally injected twice with JIB-04 (75 mg/kg of body weight) or DMSO at 24 h and 6 h prior to TGEV infection. TGEV-DMSO and TGEV-JIB-04 groups were orally infected with 1 × 10^7.25^ (1.778 × 10^7^) TCID_50_ (equivalent to 1.245 × 10^7^ PFU) of TGEV in 1.5 ml of DMEM per pig. Neonatal pigs were weighed and observed for clinical signs every 8 h throughout the study. Serum samples were collected from each pig at 24 and 48 hpi to detect specific anti-TGEV antibodies. The occurrence of diarrhea was monitored, and its severity was recorded based on an established scoring system (49). In brief, diarrhea was scored based on color, consistency, and amount and numbered as the following: 0, normal; 1, pasty; 2, semiliquid; 3, liquid. A score of ≥2 was considered diarrhea. At 48 hpi, all pigs were euthanized, and intestinal tissues were collected for pathological examination and viral load analysis using RT-qPCR.

### (ii) Therapeutic administration experiment.

A total of 19 animals were used and divided into three groups: a DMSO administration control group (control, *n* = 6), a DMSO administration and TGEV infection group (TGEV-DMSO, *n* = 7), and a JIB-04 administration and TGEV infection group (TGEV-JIB-04, *n* = 6). TGEV-DMSO and TGEV-JIB-04 groups were orally infected with the same dose of TGEV as that used in prophylactic administration experiments in 1.5 ml of DMEM per pig. At 6 hpi and 24 hpi, pigs were intraperitoneally injected twice with JIB-04 (75 mg/kg) or DMSO. Neonatal pigs were weighed and observed for clinical signs every 24 h. The occurrence of diarrhea was monitored every 8 h, and its severity was recorded as described for prophylactic administration experiments. At 48 hpi, 3 pigs from each group were euthanized, and intestinal tissues were collected for pathological examination and viral load analysis using RT-qPCR. Remaining animals continued to be monitored for survival, weight loss, and diarrhea severity for another 3 days.

### Histopathological and immunofluorescence analysis.

Intestinal tissues harvested from pigs were fixed in 4% PFA in PBS and incubated in 50% ethanol overnight. After fixation, tissues were embedded in paraffin, sectioned, and subjected to hematoxylin and eosin staining by standard procedures. For immunofluorescence analysis, samples were probed with rabbit anti-TGEV-N antibody (1:500; DA0224; Shanghai YouLong Biotech) for 30 min at 37°C. After three washes, samples were incubated with Cy3-conjugated goat anti-rabbit secondary antibody (Beyotime) and 4′,6-diamidino-2-phenylindole (DAPI) (Invitrogen). Images were obtained using a fluorescence microscope (Carl Zeiss).

### Ethics statement.

Primary human airway epithelial cells were isolated from excess surgical tissue of lungs provided by deceased donors. Cells were deidentified and exempted from regulated human subject research by the institutional review board at Washington University School of Medicine. Animal experiments were approved by the Committee on the Ethics of Animal Care and Use of the Science and Technology Agency of Jiangsu Province. The approval ID is NKYVET 2014-63, granted by the Jiangsu Academy of Agricultural Sciences Experimental Animal Ethics Committee. All efforts were made to minimize animal suffering. The virus challenge and tissue collection were performed in strict accordance with the guidelines of Jiangsu Province Animal Regulations (decree no. 2020-18).

### Statistical analysis.

All bar graphs are displayed as means ± standard errors of means (SEM). Statistical significance in data for Fig. 1D, 2D to F, 3C, 4C and D, and 5B and C and Fig. S3E was calculated by Student's *t* test using Prism 8.4.3 (GraphPad). Statistical significance in data for Fig. 1C, 2D and G, and 3B, E, F, and H and Fig. S3A and D, S4B, and S5C was calculated by pairwise analysis of variance (ANOVA) using Prism 8.4.3. Nonlinear regression (curve fit) was performed to calculate EC_50_ and CC_50_ values for Fig. 1B and 2A and Fig. S2D and S3I and J using Prism 8.4.3. Highest single agent, Bliss independence, Loewe additivity, and zero interaction potency synergy model were applied to calculate the synergy scores of dose-response data for Fig. 3A using a web application, SynergyFind v2. Gehan-Breslow-Wilcoxon test was used to compare the survival curves in Fig. 4B and Fig. S5A. All data are presented as asterisks (*, *P* ≤ 0.05; **, *P* ≤ 0.01; ***, *P* ≤ 0.001). All experiments other than Fig. 1A, 3J, 4, and 5 were repeated at least twice. The raw data are included in Table S3.

10.1128/mbio.03377-21.8 TABLE S3 Raw data. Download Table S3, XLSX file, 0.03 MB. Copyright © 2022 Son et al. 2022 Son et al. https://creativecommons.org/licenses/by/4.0/ This content is distributed under the terms of the Creative Commons Attribution 4.0 International license.

### Data availability.

The RNA-seq raw and processed data sets were deposited in the NCBI Gene Expression Omnibus database under accession number GSE156219. All raw data in the current study are available in Table S3 and Data Set S1.
