# Supplementary figures and images for: JIB-04 Has Broad-Spectrum Antiviral Activity and Inhibits SARS-CoV-2 Replication and Coronavirus Pathogenesis
Source: mBio. 2022 Jan 18;13(1):e03377-21. doi: 10.1128/mbio.03377-21 (PMC8764536; doi:10.1128/mbio.03377-21)

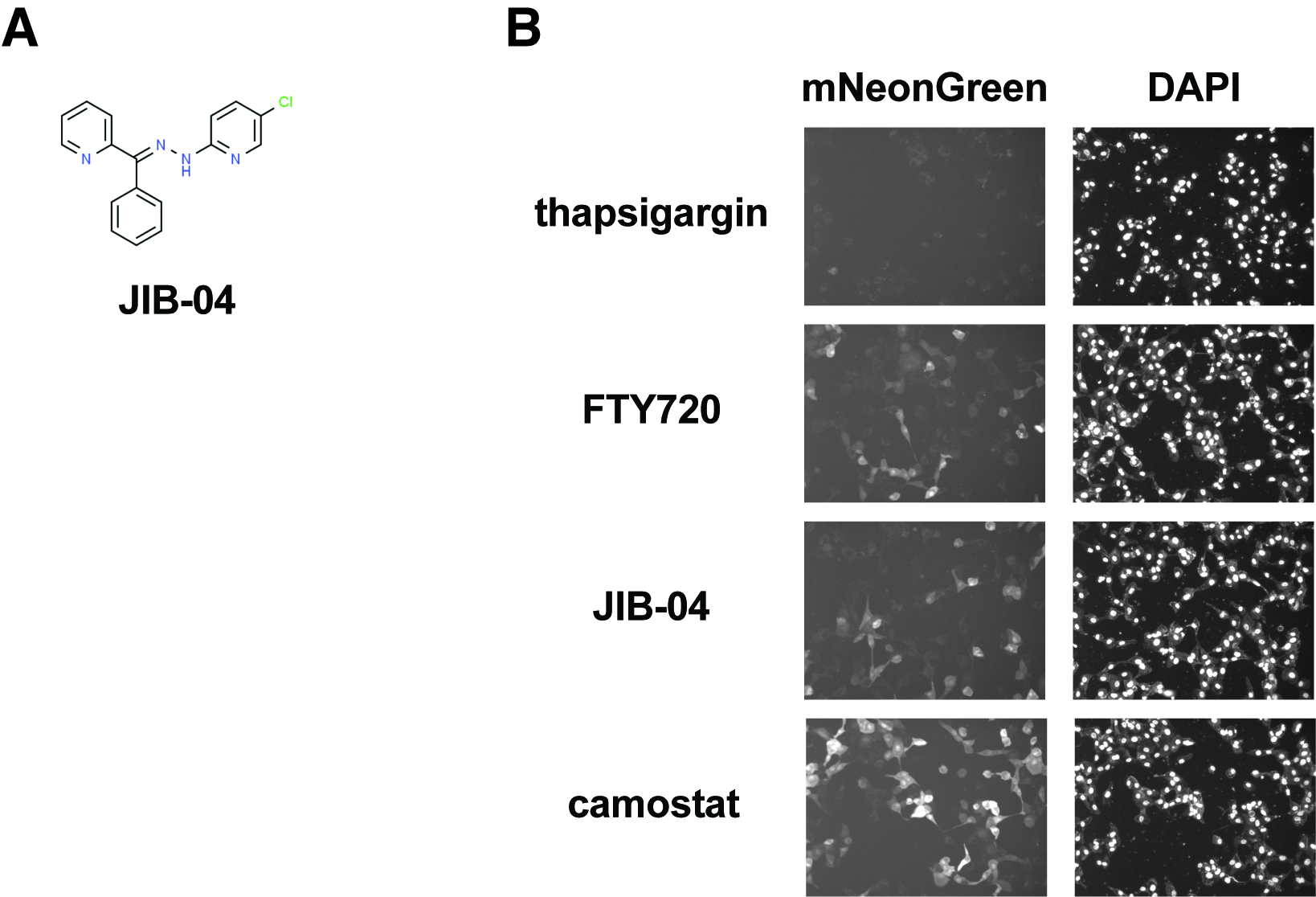

Supplement: FIG S1 [file mbio.03377-21-sf001.tif]

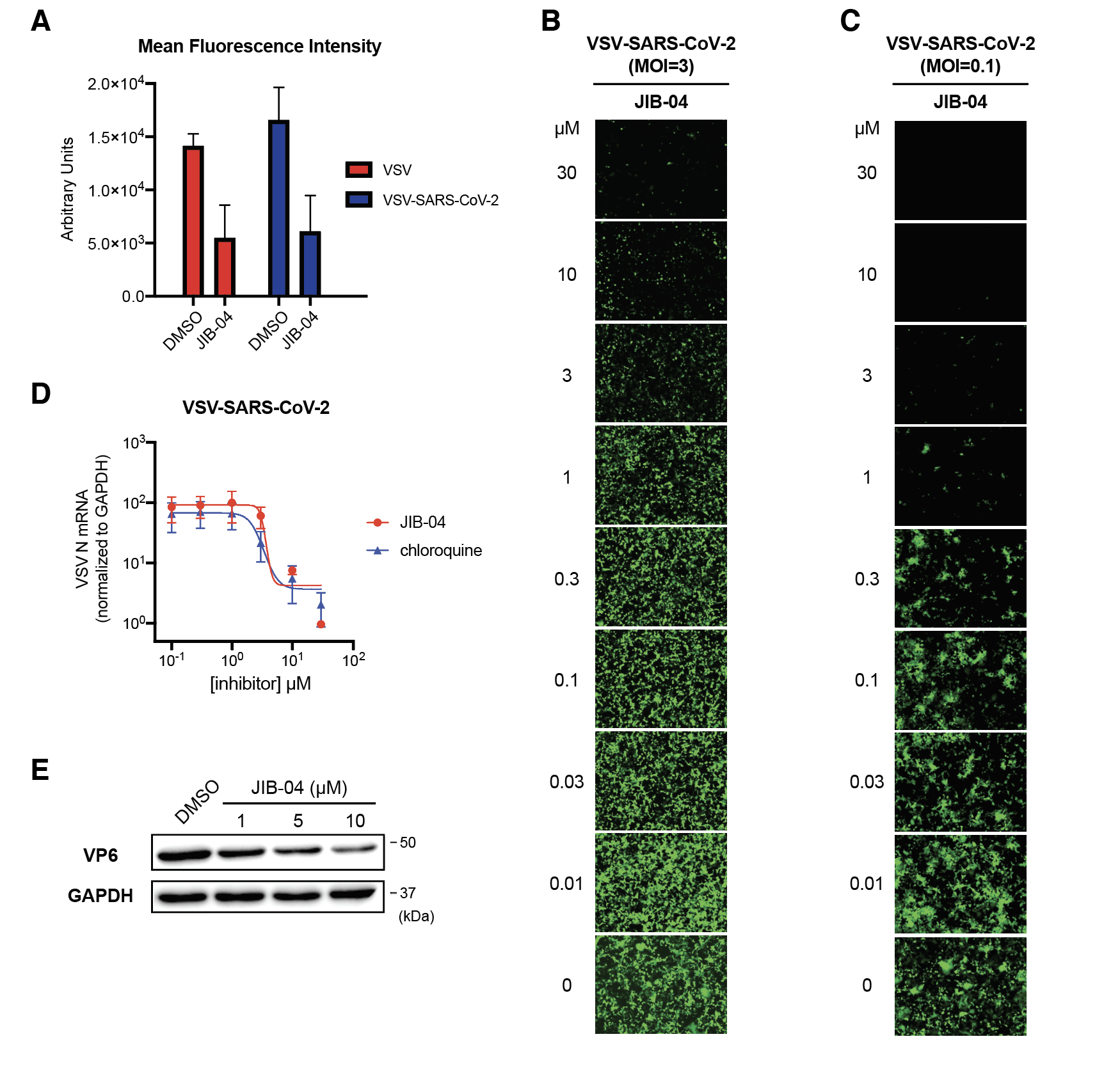

Supplement: FIG S2 [file mbio.03377-21-sf002.tif]

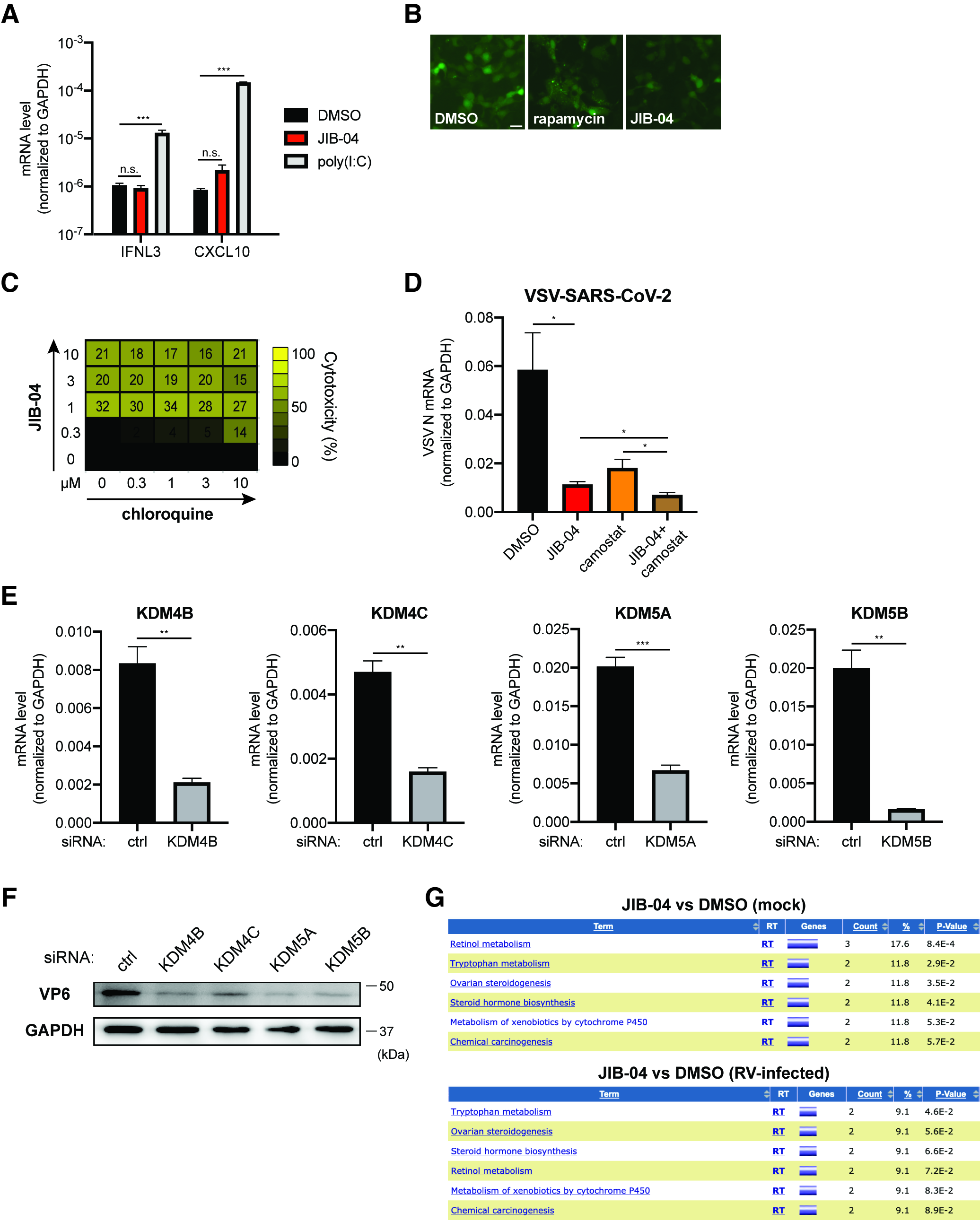

Supplement: FIG S3 [file mbio.03377-21-sf003.tif]

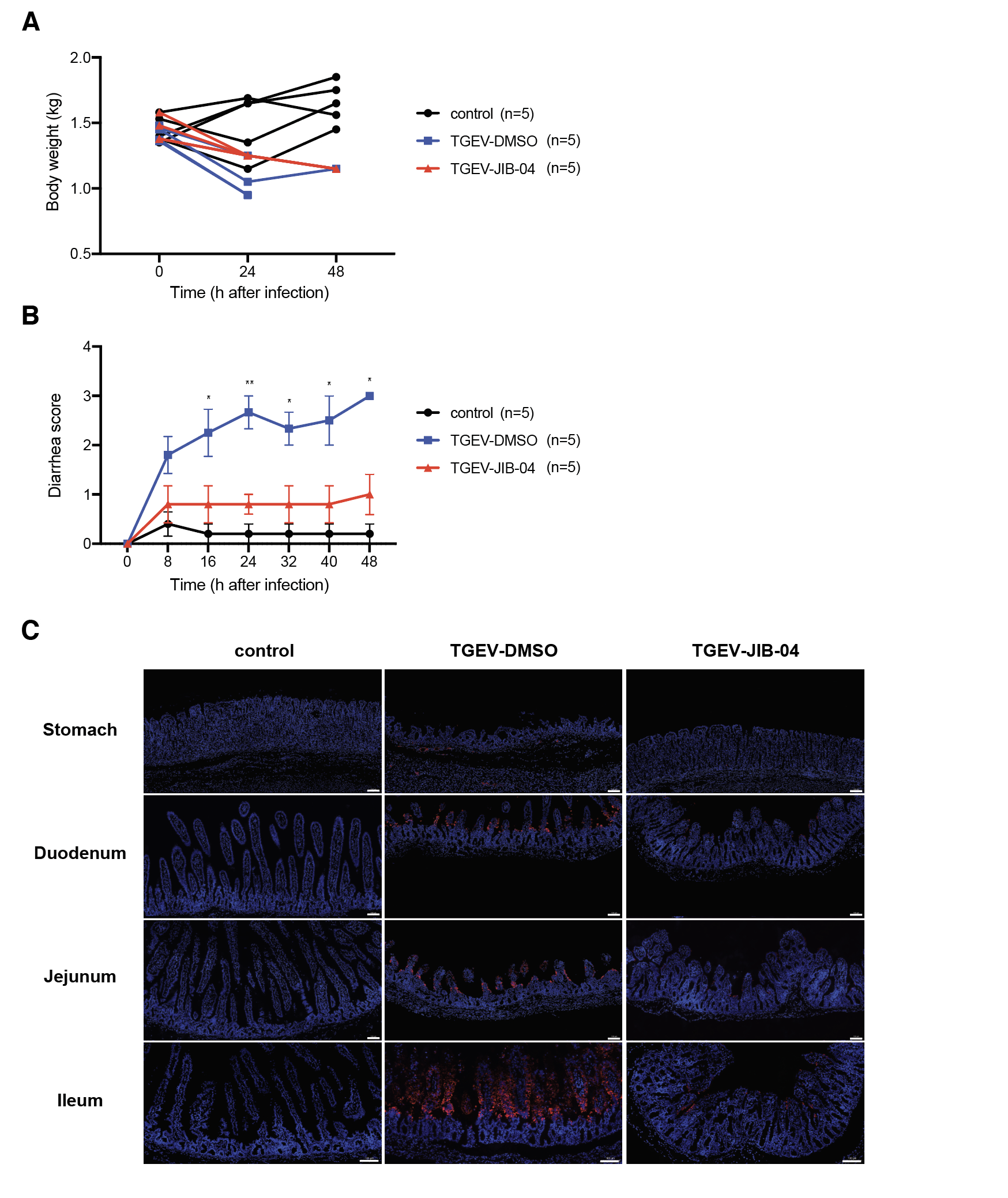

Supplement: FIG S4 [file mbio.03377-21-sf004.tif]

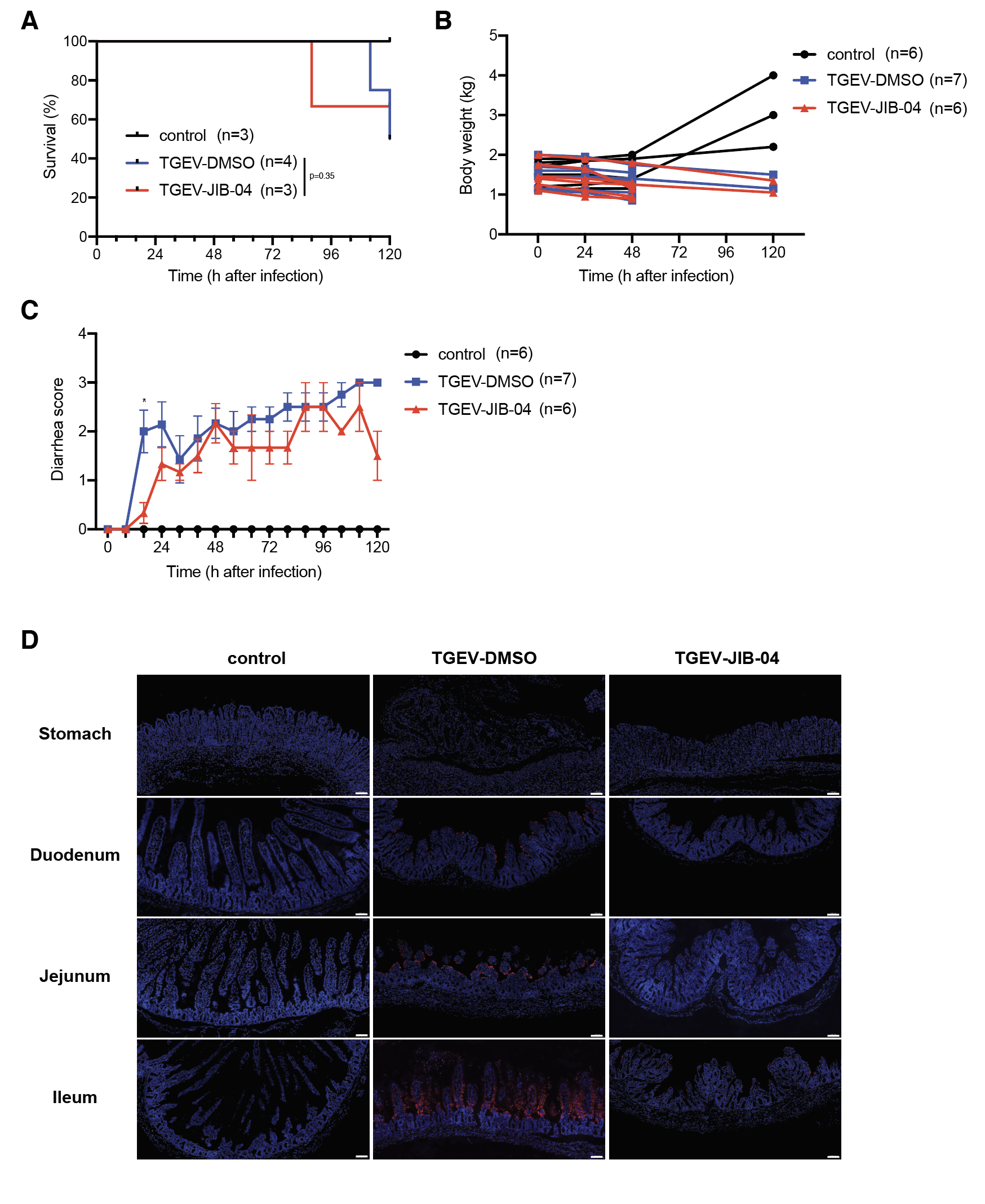

Supplement: FIG S5 [file mbio.03377-21-sf005.tif]
